# Supplementary material for: A case series shows independent vestibular labyrinthine function after major surgical trauma to the human cochlea
Source: Commun Med (Lond). 2021 Oct 6;1:37. doi: 10.1038/s43856-021-00036-w (PMC9053204; doi:10.1038/s43856-021-00036-w)
Supplement: Supplementary file 3 — Reporting Summary [file 43856_2021_36_MOESM3_ESM.pdf]

nature research

Corresponding author(s): Stefan K. Plontke

Last updated by author(s): Sep 8, 2021

Reporting Summary

Nature Research wishes to improve the reproducibility of the work that we publish. This form provides structure for consistency and transparency in reporting. For further information on Nature Research policies, see our Editorial Policies and the Editorial Policy Checklist.

Statistics

For all statistical analyses, confirm that the following items are present in the figure legend, table legend, main text, or Methods section.

n/a	Confirmed	
☐	☒	The exact sample size (*n*) for each experimental group/condition, given as a discrete number and unit of measurement
☐	☒	A statement on whether measurements were taken from distinct samples or whether the same sample was measured repeatedly
☐	☒	The statistical test(s) used AND whether they are one- or two-sided<br>*Only common tests should be described solely by name; describe more complex techniques in the Methods section.*
☒	☐	A description of all covariates tested
☐	☒	A description of any assumptions or corrections, such as tests of normality and adjustment for multiple comparisons
☐	☒	A full description of the statistical parameters including central tendency (e.g. means) or other basic estimates (e.g. regression coefficient) AND variation (e.g. standard deviation) or associated estimates of uncertainty (e.g. confidence intervals)
☐	☒	For null hypothesis testing, the test statistic (e.g. *F*, *t*, *r*) with confidence intervals, effect sizes, degrees of freedom and *P* value noted<br>*Give P values as exact values whenever suitable.*
☒	☐	For Bayesian analysis, information on the choice of priors and Markov chain Monte Carlo settings
☒	☐	For hierarchical and complex designs, identification of the appropriate level for tests and full reporting of outcomes
☒	☐	Estimates of effect sizes (e.g. Cohen's *d*, Pearson's *r*), indicating how they were calculated

Our web collection on statistics for biologists contains articles on many of the points above.

Software and code

Policy information about availability of computer code

Data collection	No software was used.
Data analysis	For all statistical calculations, GraphPad Prism software (Version 8, Graphpad Software, San Diego, CA, USA) was used.

For manuscripts utilizing custom algorithms or software that are central to the research but not yet described in published literature, software must be made available to editors and reviewers. We strongly encourage code deposition in a community repository (e.g. GitHub). See the Nature Research guidelines for submitting code & software for further information.

Data

Policy information about availability of data

All manuscripts must include a data availability statement. This statement should provide the following information, where applicable:

- Accession codes, unique identifiers, or web links for publicly available datasets
- A list of figures that have associated raw data
- A description of any restrictions on data availability

Source data for the main figures in the manuscript can be accessed as Supplementary Data 1. The remaining data cannot be made publicly available because the ethical approval and the informed consent from the patients included in this study did not cover placing excerpts / copies from patient charts like written records of medical history, or complete electrophysiological curves like full VEMP-, vHIT-, or audiogram traces etc., or third party written reports or images (e.g. MRI, histology etc.) into publicly open repositories. Relevant portions of those data can be accessed from the authors upon relevant ethical approval by contacting the corresponding author on reasonable request.

Field-specific reporting

Please select the one below that is the best fit for your research. If you are not sure, read the appropriate sections before making your selection.

☒ Life sciences ☐ Behavioural & social sciences ☐ Ecological, evolutionary & environmental sciences

For a reference copy of the document with all sections, see nature.com/documents/nr-reporting-summary-flat.pdf

Life sciences study design

All studies must disclose on these points even when the disclosure is negative.

Sample size	The entity is a (very) rare disease. ALL patients with this disease entity and disease extent (intracochlear tumor location only) who were surgically treated in our institution between 2011 and 2020 were inlcuded.
Data exclusions	No data were excluded from the analysis.
Replication	n/a
Randomization	The study was a one-arm observational study.
Blinding	The study was a one-arm unblinded observational study.

Reporting for specific materials, systems and methods

We require information from authors about some types of materials, experimental systems and methods used in many studies. Here, indicate whether each material, system or method listed is relevant to your study. If you are not sure if a list item applies to your research, read the appropriate section before selecting a response.

Materials & experimental systems

n/a	Involved in the study	
☒	☐	Antibodies
☒	☐	Eukaryotic cell lines
☒	☐	Palaeontology and archaeology
☒	☐	Animals and other organisms
☐	☒	Human research participants
☐	☒	Clinical data
☒	☐	Dual use research of concern

Methods

n/a	Involved in the study	
☒	☐	ChIP-seq
☒	☐	Flow cytometry
☒	☐	MRI-based neuroimaging

Human research participants

Policy information about studies involving human research participants

Population characteristics	27 patients (29–75 years, 13 male, 14 female) with solely intracochlear schwannomas. No (other/non disease-related) neurological disorders.
Recruitment	Patients included all consecutive patients with surgical resection of intracochlear schwannomas, most of them with hearing rehabilitation with a cochlear implant in a single tertiary university referral center.
Ethics oversight	Ethics Committee of the Martin Luther University Halle-Wittenberg / University Medicine Halle, Germany

Note that full information on the approval of the study protocol must also be provided in the manuscript.

Clinical data

Policy information about clinical studies

All manuscripts should comply with the ICMJE guidelines for publication of clinical research and a completed CONSORT checklist must be included with all submissions.

Clinical trial registration	There is no clinical trial registration (monocentric case series).
Study protocol	The study comprises a large retrospective and prospective monocentric case series analyzing treatment results from a tertiary (university) referral center. The study protocol as submitted to the Ethics committee is availale on request from the authors.
Data collection	Setting: single tertiary university referral center; Time period od recruitment and data collection: 2011 to 2020

Outcomes	A detailed description of the outcome measures has been reported in the "Methods" section of the manuscript.
